# Neurodevelopmental outcomes for pediatric ventricular assist device and heart transplant patients in the modern Era

**DOI:** 10.3389/fcvm.2026.1889471

**Published:** 2026-08-10

**Authors:** Shannon Oliver, Holger Buchholz, Darren H. Freed, De Villiers Jonker, Ari R. Joffe, Simon Urschel, Tara Pidborochynski, Joseph Atallah, Charlene M. T. Robertson, Jessica Zvonkovic, Jennifer Conway

**Affiliations:** 1 Department of Pediatrics, University of Alberta, Edmonton, Alberta, Canada; 2 Department of Cardiac Surgery, University of Alberta, Edmonton, Alberta, Canada; 3 Department of Pediatrics, Glenrose Rehabilitation Hospital, Complex Pediatric Therapies Follow-up Program, Edmonton, Alberta, Canada

**Keywords:** heart transplant, outcomes, pediatric, review, ventricular assist device

## Abstract

The majority of patients who undergo pediatric ventricular assist device implantation or heart transplant are expected to survive to adulthood. However, there is limited research into what their neurodevelopmental and neurocognitive trajectories will be. This narrative mini review summarizes the literature available, demonstrating the significant burden of neurodevelopmental and neurocognitive difficulties in survivors, and highlights the importance of improving anticoagulation and developmental care in this population aiming for patients to reach their full potential. There is ongoing need for further research, and collaboratives such as the Advanced Cardiac Therapies Improving Outcomes Network can play an integral role in facilitating our understanding of the risk factors and long-term outcomes in this complex and high-risk patient population.

## Introduction

Heart Transplant (HTx) is the gold standard of care for children with end stage heart failure (1), and ventricular assist device (VAD) are part of the standard management of advanced heart failure (2). Registries such as Advanced Cardiac Therapies Improving Outcomes Network (ACTION), Pediatric Heart Transplant Society (PHTS), and the Pediatric Interagency Registry for Mechanical Circulatory Support (PediMACS) collect data on post VAD and post HTx survival and adverse outcomes. Recent reports from these registries suggested that the majority of patients undergoing either VAD or HTx will survive, with PediMACS reporting that by 12 months post VAD implant, 75% of patients will have successfully undergone HTx, or been decannulated due to recovery (with 9% of patients still alive on a device) (3). For patients who undergo HTx before 10 years of age, 10 year survival (conditional on surviving 1 year post HTx) is reported at 83%, with overall survival to 10 years post HTx being 70% and 68% for cardiomyopathy patients and congenital heart disease (CHD) patients respectively (1).

**Figure 1:**
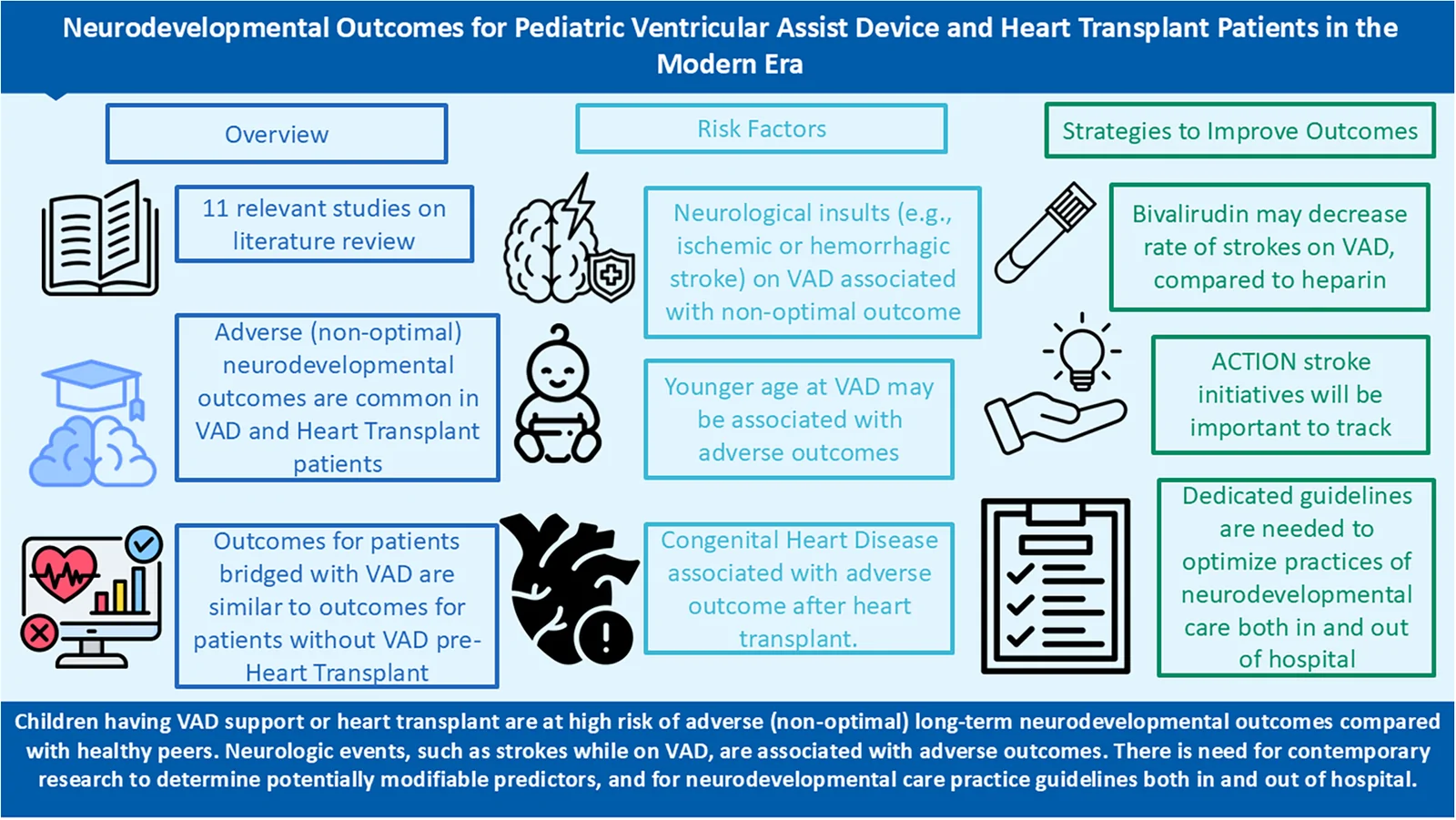
This narrative review highlights the significant burden of, risk factors for, and potential strategies to mitigate adverse outcomes in pediatric ventricular assist device and heart transplant patients.

In the context of these survival outcomes, it is important to also focus on life post VAD and HTx. This includes understanding the neurodevelopmental trajectory for these children. This narrative review article aimed to highlight the burden of neurodevelopmental, neurocognitive, and sensorimotor adverse outcomes in the pediatric VAD and HTx populations, and to provide information as to what strategies are in place to continue to improve these outcomes (Figure 1). This was done via a literature review of the PubMed database using the terms “neurodevelopmental” OR “neurocognitive” AND “heart transplant” OR “ventricular assist device”, limited to children up to 18 years of age and to the past 5 years, searched to March 2026. The search retrieved 498 titles, from which 11 titles were retained in the narrative review, and some relevant older references from these titles were also included.

## Commonly used neurodevelopmental and neurocognitive assessments in the VAD and HTx outcomes literature

Neurodevelopmental and neurocognitive assessments are performed by health practitioners trained to administer age specific tests. For children who are 1–43 months of age, one of the more commonly used tools for outcomes assessment in pediatric HTx and VAD literature is the Bayley Scales of Infant and Toddler Development III or IV (Bayley-III or Bayley-IV). These are used to assess cognitive, language (receptive and expressive language), and motor skills (gross and fine motor) (4, 5). For kindergarten aged children, formal cognitive testing is performed, usually using the Wechsler Preschool and Primary Scales of Intelligence (WPPSI) Third (WPSSI-III) or Fourth (WPSSI-IV) edition, from which determination can be made of the Full Scale Intelligence Quotient (FSIQ) (6, 7), and often the Beery-Buktenica Developmental Test of Visual-Motor Integration (VMI) (Fifth or Sixth edition) (8, 9) is used to assess nonverbal integration of visual and motor skills. In addition to the aforementioned assessments, skills required for day-to-day functioning can be assessed, usually with the Adaptive Behavior Assessment System (ABAS-3) (10), including determination of the General Adaptive Composite (GAC) score. There are many other standardized assessment tools that can be used to determine outcomes, including tools for executive function, behavior, and adaptive functioning; review of these is beyond the scope of this paper.

One of the challenges with scores such as the Bayley-IV or the WPSSI-IV is that they only assess and report neurodevelopmental or neurocognitive domains, and do not necessarily encapsulate the outcome of the child as a whole. In particular, they may not fully account for sensorimotor disabilities that may impact a child's overall functional trajectory, for example cerebral palsy or hearing deficits. In 2023, Khoury et al. defined the concept of an optimal neurodevelopmental outcome, being a composite outcome that takes into consideration both a child's scores on standardized, age appropriate testing (i.e., scores of ≥80, that is, at the 9th percentile or higher of population norms, indicating low average or above scores according to the American Psychological Association), and the presence of any disability including seizure disorder, cerebral palsy (or later acquired motor impairment), or permanent severe vision or hearing loss (11). This was a novel way of reporting outcomes, rather than reporting individual standard scores (with standard deviation ranges) and sensorimotor disability separately. This concept has subsequently been utilised in both the CHD population and the post VAD population, with the idea of aiming to give a more global sense of a child's neurodevelopmental outcome (11–13). As with any outcome assessment, reporting outcomes as optimal/non-optimal has limitations, especially that such a dichotomous outcome may misclassify patients because some children may have important adverse outcomes not included or measured (e.g., in behavior, emotional health, social skills, executive function, etc.), and others may have a good quality of life while being classified as having a non-optimal outcome.

## Neurodevelopmental, neurocognitive, and sensorimotor outcomes in VAD and HTx patients

Historically, there had been limited research into neurodevelopmental outcomes for children undergoing VAD implantation. Rather, the focus had been on neurodevelopmental outcomes and quality of life (QoL) assessments post HTx (14–18). However, two recent studies examined neurodevelopmental outcomes in the pediatric VAD population in Western Canada, both suggesting that VAD implantation in early childhood was associated with a significant burden of neurodevelopmental impairments (12, 13). When examining factors associated with adverse neurodevelopmental outcomes, both showed that patients with neurological insults while on a VAD (in particular, ischemic stroke, hemorrhagic stroke, and/or extra-axial intracranial bleeding) were more likely to have a non-optimal outcome (12, 13). The adjusted odds ratio (aOR) after neurological insult for optimal outcome was 0.10 [95% confidence interval (CI) 0.01–0.63], *p* = 0.012, in children implanted with a VAD at <6 years of age, and for non-optimal outcome the aOR was 12.35 [95% CI =  1.29–1,660.36], *p* = 0.026, in children implanted with a VAD at ≤15 months of age (12, 13) Interestingly, there was no statistically significant difference in the proportion of patients with or without CHD in children with optimal (and non-optimal) neurodevelopmental outcome (12, 13). This is important, as multiple studies have demonstrated that children with CHD are at higher risk of adverse neurodevelopmental outcomes compared to children with structurally normal hearts (19–21). This suggests that factors other than CHD are at play impacting neurodevelopmental outcomes after VAD support. These findings are supported in a recent paper using the United Network for Organ Sharing (UNOS) data to examine the motor and cognitive trajectory in patients bridged to HTx with a VAD (22). Patients experiencing a stroke while being bridged to HTx with VAD or extracorporeal membrane oxygenation (ECMO) were more likely to have progression of neurodevelopmental delay from pre- to post-transplant (aOR = 2.39, 95% CI = 1.13, 5.02; *p* = 0.021) (22). Moreover, CHD was not an independent predictor of progression of neurodevelopmental delay (aOR = 1.48, 95% CI = 0.97, 2.25; *p* = 0.069), further suggesting that it is not only a comorbid CHD diagnosis impacting neurodevelopmental outcomes after VAD support (22). Even within a large cohort of young children with CHD, extracorporeal support was strongly and independently associated with adverse neurocognitive and functional outcomes (19).

The UNOS data also demonstrated that younger patients who were bridged to HTx with VAD were more likely to exhibit progression of neurodevelopmental and neurocognitive delay (22). This aligns with the findings by Oliver et al., where three-quarters of children implanted with a VAD at ≤15 months of age and surviving to assessment age had a non-optimal neurodevelopmental outcome at 2 years of age (12), and two-thirds of children implanted with a VAD at <6 years of age and surviving to assessment age had a non-optimal outcome at 6 years of age (13).

For the HTx population, both Uzark et al., and Fleisher et al. demonstrated that pediatric HTx recipients were at risk of neurodevelopmental delay, particularly with regards to language (16, 17). It is worth noting that these studies relied on historical data, with recipients in the Uzark et al. study undergoing HTx between 1996 and 2006, and in the Fleisher et al. study between 1984 and 2000 (16, 17). With advances in the field of pediatric HTx since that era, one might hypothesize that these findings are not as relevant in a more contemporary era. However, in a more recent analysis of recipients who underwent HTx from 1999 to 2011, Urschel et al. demonstrated that children who undergo pediatric HTx have a mean FSIQ, VMI, performance intelligence quotient (PIQ) and verbal intelligence quotient (VIQ) shifted to the left (below) of population norms (18). In particular, they reported that for children who underwent HTx in the context of CHD, 40% had an FSIQ < 70 (more than 2 standard deviations below population norms, expected in 2.27% of the normative population) (18). This suggested that while the advances in care may have led to improved post HTx survival (23), neurodevelopmental and neurocognitive outcomes remain a significant concern. In this study, VAD utilization before transplantation (in *n* = 15 of 55 HTx children with outcomes determined) was not independently associated with different neurocognitive outcomes; however, prolonged in-hospital and intensive care courses and underlying CHD were strong independent predictors of lower neurocognitive performance (18).

There are two other studies specifically examining the impact of pre-HTx VAD on post-HTx cognitive and neurodevelopmental outcomes (24, 25). The study by Stein et al. spanned an era from 1984 to 2010 and compared cognitive outcomes between patients who did (*n* = 9) and did not (*n* = 11) receive VAD while awaiting HTx (24). This study did not perform statistical tests for differences in cognitive outcomes because the cohorts were too small, although there was visual overlap of the 95% standard error bars on each outcome (24). A more recent study by James et al., from 2017 to 2022, demonstrated on univariate analysis that there was no statistically significant difference between the proportion of patients with neurodevelopmental delay post-HTx between those on pre-HTx VAD vs. no pre-HTx VAD (12/13, 92% vs. 22/24, 92% *p* = 1.000), although numbers were very small (25). The authors additionally reviewed neurodevelopmental outcomes of their whole HTx cohort in that era, and, in keeping with studies by Urschel et al., Uzark et al., and Fleisher et al., they demonstrated a high burden of morbidity, with 93% of patients having neurodevelopmental delay (16–18, 25).

A lack of differences in Stein et al. was postulated to be due to VAD supporting end organ function, thereby providing a degree of neuroprotection, despite the previously mentioned known risks (24). Conversely, James et al. hypothesized that pre-HTx VAD did not confer an advantage due to the patients already being quite unwell at time of implant (25). Both these theories could be supported by the fact that children with advanced heart failure may be in a state of cerebral hypoperfusion, and at risk of neurological complications secondary to this. Therefore, early implantation of a VAD may help mitigate these risks while awaiting HTx (26). Alternatively, these studies could have been biased by the fact that many patients had a comorbid CHD diagnosis. In the James et al. study, two-thirds of their patients had a CHD diagnosis (25), while for Stein et al., 27% of patients without VAD had CHD compared with only 11% of patients who had VAD support (24). This could be significant because, as previously mentioned, CHD is known to be a risk factor for worse neurodevelopmental outcomes post HTx (18). As VAD therapy is becoming more frequent, given the known neurodevelopmental sequelae associated with VAD support, it will be important to see in larger studies what the overall impact of VAD support pre-HTx is on neurodevelopmental outcomes. With small cohorts in the reviewed studies, it has been difficult to control for severity of illness at the time of VAD implantation, and for propensity to implement VAD support, making determination of any contribution of VAD support to either worse or better long-term neurodevelopmental, neurocognitive, or disability outcomes uncertain.

## Discussion

### Improving outcomes

A common theme noted in the studies investigating neurodevelopmental outcomes post VAD was the negative impact of neurological insults while on a VAD. Stroke while on a VAD is a well described complication, typically due to thromboembolic events or intracranial hemorrhage associated with anticoagulation (27–29). There has been a concerted effort in the VAD community to reduce the rates of stroke while on VAD. The “ABC's of Stroke Prevention” initiative through ACTION focused on blood pressure control, anticoagulation, and communication between team members as a way to decrease the rates of strokes in the pediatric VAD population (27). One of the key focuses of this ACTION initiative was to improve anticoagulation. Several observational studies have demonstrated that switching from unfractionated heparin to bivalirudin has been associated with a decrease in rates of stroke (13, 28, 29), with Adderley et al. demonstrating a hazard reduction [Hazard Ratio (HR) 0.30 (95% CI = 0.12–0.75), *p* = 0.01] in survival free from thromboembolic events (29). In an analysis of PediMACS data, patients implanted with a VAD prior to 2017 were at a higher risk for stroke compared to those implanted afterwards [HR = 2.10 (95% CI = 1.40– 3.15), *p* = 0.030] (30). While this era analysis did not specifically examine anticoagulation strategies, as use of bivalirudin began in 2015, it may be that the improvement in stroke rates post 2017 are at least partly attributable to changes in anticoagulation strategies (30). Interestingly, bivalirudin is the only direct antithrombin inhibitor shown to be associated with a lower stroke rate, with a small study into argatroban not demonstrating this association compared to heparin (31).

Oliver et al. demonstrated the interesting finding, in their 2026 study, that 12% of infants who had a non-optimal neurodevelopmental outcome at 2 years of age, improved to have an optimal outcome when reassessed at 4.5–7 years of age (13). This suggested that it is possible for a child's neurodevelopmental trajectory to improve over time post VAD. Similarly, Chinnock et al. found improving neurocognitive abilities with older age and longer time post-transplant especially in the context of specific learning support (32). This would be in keeping with the known overall concepts of neurodevelopmental care and early intervention, irrespective of cardiac disease (33).

Guidelines and scientific statements for children with CHD emphasize the importance of early assessment and intervention aiming to optimize neurodevelopmental outcomes for this cohort (34–36). A recent guideline from the American Heart Association acknowledged that children with CHD having HTx and/or VAD support are in a “high risk” category for adverse outcomes and require attention to prompt referral for early intervention (34). Even so, there are no consensus statements for neurodevelopmental care specifically for pediatric VAD or HTx populations. In an attempt to rectify this, the Cardiac Neurodevelopmental Outcome Collaborative and ACTION have created a collaboration to enhance neurodevelopmental understanding and resources for patients on VAD (37). They completed an initial survey across ACTION sites which demonstrated considerable variability of practice, with only 28.8% of centers offering a culture in which there was a consistent approach to supporting early development in VAD patients, and 45% of centers relying on treating clinicians to determine whether the child should be referred for assessment, as opposed to there being a blanket referral pathway for pediatric VAD patients (37). This study served to highlight the ongoing need for research into neurodevelopmental trajectories post VAD implant, and the importance of quality improvement initiatives to improve neurodevelopmental care in this vulnerable population (37).

The need for, and potential of, neurodevelopmental care in children in intensive care environments cannot be over-emphasized (38). In addition, we suggest that structured long-term follow-up of patients treated with VAD should be a standard of care. The studies by Oliver et al. demonstrated the importance of such follow-up programs as the Complex Pediatric Therapies Follow-up Program in western Canada (12, 13). This type of follow-up, aiming to provide service (by detecting problems and implementing early intervention as indicated for the individual child) and quality improvement (by determining potentially modifiable acute-care predictors of long-term outcomes that iteratively inform care over time) are necessary if patients having VAD support are to achieve their full potential and we are to improve management over time (39). Future multicenter collaborations will be necessary to achieve the sample sizes needed to robustly inform practice.

### Limitations

This narrative review has limitations. The main limitation is that this was not a systematic review, and relevant publications may have been missed. Nevertheless, we believe that the goal of giving an overview of neurodevelopment in children after VAD or HTx was achieved. The information reviewed was observational and often from single centers, and inferences from reported associations are hypothesis generating as residual confounding cannot be ruled out. Finally, most studies reported outcomes in younger children, and generalization to older children should be done with caution.

## Conclusions

There are currently limited studies evaluating the impact of VAD and HTx on the neurodevelopmental trajectories for patients who undergo these interventions in childhood. The available evidence suggests that these children are at high risk compared to their healthy peers. Adverse events such as strokes on VAD have been consistently demonstrated to be associated with worse neurodevelopmental outcomes. An emphasis on improving anticoagulation may lead to better outcomes in the modern era. Learning networks such as ACTION will play an invaluable role in being able to gain a better understanding of practices across centers, and to enable the development of standardized guidelines for neurodevelopmental care and follow-up for VAD patients. This review also highlights the fact that there is a need for more contemporary research into the neurodevelopmental outcomes and associated modifiable predictors for this cohort of patients, and for a better understanding of the interplay between VAD and HTx on these outcomes.
